# Bronchiolar adenoma with EGFR exon 19 deletion mutation: a case report and literature review

**DOI:** 10.3389/fonc.2025.1565549

**Published:** 2025-04-09

**Authors:** Shaobo Zhang, Xiaohui Liu, Xiaoxia Zhang, Peng Zhang, Haiming Feng, Peng Jiang

**Affiliations:** ^1^ Department of Thoracic Surgery, The Second Hospital and Clinical Medical School, Lanzhou University, Lanzhou, China; ^2^ Department of Burns and Plastic Surgery and Wound Repair Surgery, The Second Hospital and Clinical Medical School, Lanzhou University, Lanzhou, China; ^3^ Department of Clinical Laboratory, The Second Hospital and Clinical Medical School, Lanzhou University, Lanzhou, China; ^4^ Department of Pathology, The Second Hospital and Clinical Medical School, Lanzhou University, Lanzhou, China

**Keywords:** case report, bronchiolar adenoma (BA), mixed ground-glass opacity (mGGO), epidermal growth factor receptor (EGFR), gene mutation

## Abstract

**Background:**

Bronchiolar adenoma (BA) is a benign lung tumor characterized by nodular proliferation of bilayered bronchiolar-type epithelium with a continuous basal cell layer. The genetic characteristics of BA are not well understood. However, mutations commonly associated with lung adenocarcinoma, such as BRAF V600E, epidermal growth factor receptor (EGFR) mutations, and ALK rearrangements, have recently been identified in this context.

**Case report:**

This report describes a case of BA in a 43-year-old male who presented with a mixed ground-glass opacity (mGGO) detected during a routine physical examination. The patient had excellent cardiopulmonary function and no other medical conditions. After evaluation, local surgical resection was performed. Intraoperative frozen section pathology initially suggested adenocarcinoma. However, postoperative immunohistochemical examination confirmed the diagnosis of BA. Next-generation sequencing (NGS) further revealed an EGFR exon 19 deletion mutation.

**Conclusion:**

The histological morphology of highly differentiated small invasive adenocarcinomas and microinvasive adenocarcinomas closely resembles that of BAs in intraoperative frozen sections. Additionally, gene mutations linked to adenocarcinoma have been identified in BAs. The potential relationship between these two diseases warrants further investigation.

## Introduction

A group of benign lesions originating from the peripheral bronchial epithelium or undifferentiated tumors with malignant potential, such as bronchial epithelial dysplasia, adenoid papilloma, and ciliated muconodular papillary tumor (CMPT) (1), has caused diagnostic confusion due to their morphological variability and low incidence. Bronchiolar adenoma (BA) was first defined in 2018 by Chang et al. (2) and is categorized into proximal and distal types based on histological and immunophenotypic characteristics. The rarity of BAs has hindered the full understanding of their histogenesis and molecular features, which bear a striking resemblance to those of adenocarcinoma. This similarity has contributed to misdiagnoses during intraoperative frozen section pathology examinations. Genetic mutations commonly seen in lung cancers, including BRAF V600E (2–6), EGFR exon (2, 4, 7), KRAS (2, 6), and ALK rearrangements (8, 9), have also been identified in BAs. These findings suggest that BAs may have certain malignant attributes rather than being entirely benign. This report presents a case of BA with an EGFR exon 19 deletion mutation, which is commonly associated with non-small cell lung cancers (NSCLCs).

## Case report

**Figure 1:**
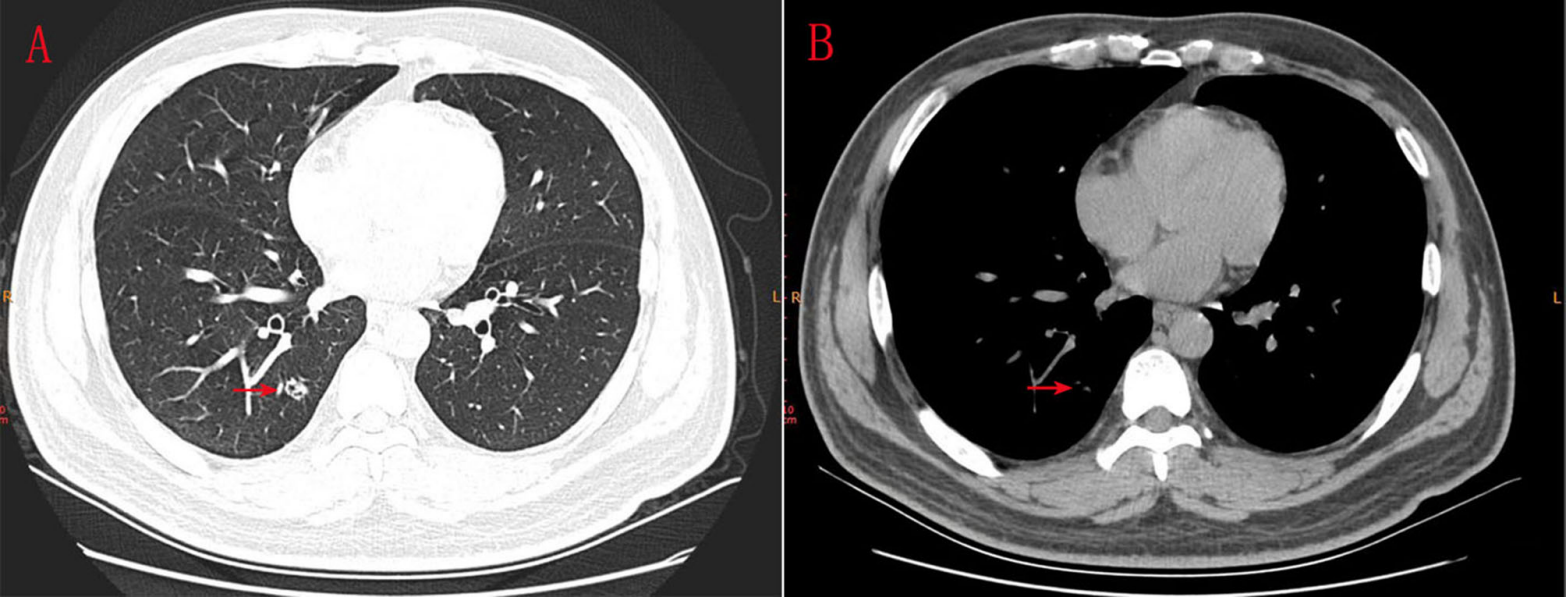
**(A, B)** CT scanning indicated an 9.7*1.1 millimeters mGGO in the posterior basal segment of inferior lobe of right lung.

**Figure 2:**
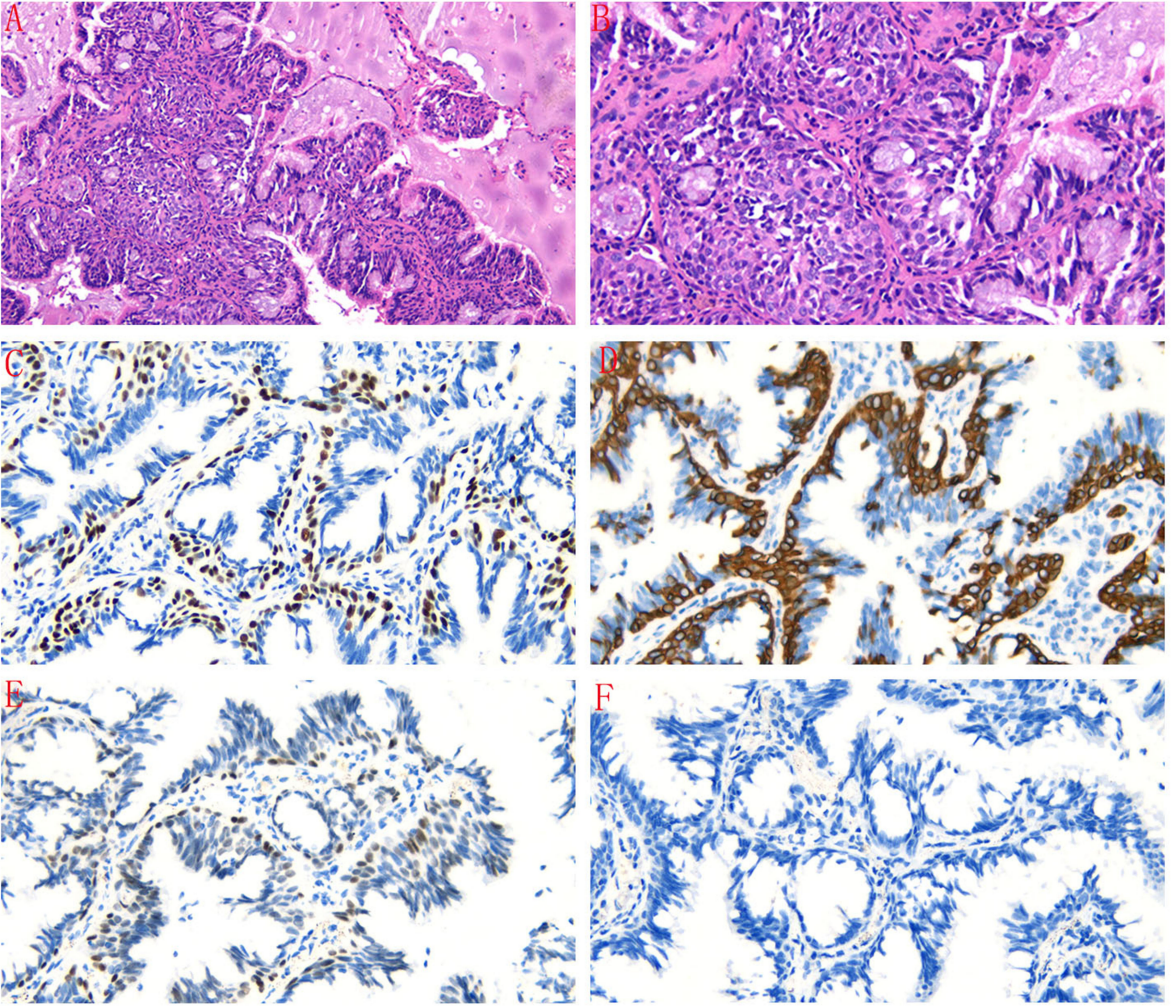
**(A, B)** The tumor tissue exhibited papillary growth, forming a double-layered cellular structure with basal and luminal cell layers. The luminal cell layer was composed of ciliated columnar cells and mucus cells (HE staining, magnification×100 and magnificatio×200). **(C)** Immunohistochemical staining showed positive expression of P63 in basal cells (magnification×200). **(D)** CK5/6 was positively expressed in basal cells (magnification×200). **(E)** TTF-1 was focal weak positive in basal cells (magnification×200). **(F)** NapsinA was negative in tumor cells (magnification×200).

A 43-year-old male presented with a mixed ground-glass nodule detected during a routine physical examination. Chest computed tomography (CT) revealed a posterior basal segment nodule measuring approximately 9.7 × 1.1 millimeters in the right lung (**Figures 1A, B**). No significant enlargement of mediastinal or hilar lymph nodes was observed. The patient’s vital signs were within normal limits. Echocardiography, abdominal ultrasonography, and pulmonary function tests all yielded normal results. Laboratory tests, including complete blood counts, serum lung cancer tumor markers and biochemical markers, were within the reference ranges. The patient did not exhibit any clinical symptoms such as cough or fever. The patient had no smoking history or family history of lung cancer. Following the diagnosis of mGGO, the patient experienced severe anxiety. After completing preoperative evaluations, the patient underwent wedge resection of the lower lobe of the right lung via video-assisted thoracoscopic surgery (VATS). Intraoperative frozen section pathology initially suggested adenocarcinoma. However, histological examination after surgery revealed that the tumor tissue displayed papillary growth, forming a double-layered cellular structure consisting of basal and luminal cell layers. Hematoxylin-eosin (HE) staining showed the luminal cell layer was composed of ciliated columnar cells and mucus cells (**Figures 2A, B**). Immunohistochemical (IHC) staining showed positive expression of P63 (**Figure 2C**), CK5/6 (**Figure 2D**) and focal weak positivity of thyroid transcription factor-1 (TTF-1) (**Figure 2E**) in basal cells. NapsinA (**Figure 2F**), Synaptophysin (Syn), CD56, and chromogranin A (CgA) were negative. Based on the strong positivity for P63, P40 and CK5/6 in the basal cell layer, which confirms the preservation of a continuous basal cell architecture, and the presence of ciliated columnar cells and mucus cells in the luminal cell layer, a diagnosis of proximal-type BA was established. Molecular pathology analysis using next-generation sequencing (NGS), a high-throughput DNA sequencing technology, revealed the presence of an exon 19 deletion mutation in the epidermal growth factor receptor (EGFR), Furthermore, no additional mutations were detected in genes such as ROS1 or ALK gene rearrangements. Two years after surgery, the patient remains free of disease recurrence.

The samples were tested in duplicate by the Department of Pathology, Second Hospital of Lanzhou University (Lanzhou, China), and Burning Rock Dx Company. The latter results were confirmed by a Clinical Laboratory Improvement Amendments (CLIA)-certified laboratory in the United States.

## Discussion

Bronchiolar adenomas (BAs) are benign lung tumors derived from bronchiolar epithelial cells, characterized by a bilayered architecture and bronchiolar differentiation (1, 10). As early as 1953, Felton introduced the concept of bronchial adenomas (BA), characterized as “peripheral, multiple” neoplasms originating from bronchial epithelial cells (11). In 2002, Ishikawa initially introduced the concept of CMPT, providing a comprehensive description and nomenclature of the tumor based on its morphological characteristics. The three cellular components and papillary structure were identified as key diagnostic features of CMPT (10). In 2017, Chang initially introduced the concept of classical and non-classical CMPT at the USCAP Annual Meeting, detailing their morphological characteristics, genetic alterations, and bronchial-like differentiation (12). In 2018, Chang further expanded this concept through a study of 25 cases, proposing the diagnostic term BA based on comprehensive morphological, immunophenotypic, and genetic analyses. Based on their structural similarity to normal bronchiolar composition, bronchioloalveolar areas (BAs) are categorized into proximal and distal types (2). Proximal-type BAs are distinguished by their papillary architecture with bilayered bronchiolar-type epithelium, including a continuous basal cell layer. The luminal cells consist of ciliated cells and mucous cells, and extracellular mucus is commonly observed in the glandular cavity. Distal-type BAs share similar cellular compositions but differ in morphology, displaying either focal or absent papillary architecture and a flat glandular cavity dominated by luminal cells. In our case, the tumor tissue exhibited a papillary architecture with bilayered glandular structures. The luminal cells were composed of ciliated cells and mucous cells, with a continuous basal cell layer. Immunohistochemical staining showed positive expression of P63, P40 and CK5/6. Based on these pathological features, a diagnosis of proximal-type BA was established.

Genetic mutations identified in BAs, including BRAF V600E, EGFR exon 19 deletions, EGFR exon 20 insertions, EGFR exon 21 p.L858R, KRAS, and ALK rearrangements, have been reported in recent years and are summarized in **Table 1** (2–9). The BRAF V600E mutation, often associated with carcinoma *in situ*, primarily indicates tumor potential. This mutation is commonly observed in neoplastic diseases such as thyroid tumors, intestinal tumors, and non-Hodgkin lymphomas, especially papillary thyroid cancer. Although it has a 2.2% incidence rate in lung adenocarcinoma, BRAF V600E is the most frequent driver mutation in BAs (2, 3, 5–7, 13, 14). KRAS mutation is a common driver mutation in NSCLC, particularly in tobacco-related cases, accounting for approximately 28% of cases. KRAS mutations represent the second most frequent driver mutation in BAs (2, 6). EGFR mutations are prevalent in advanced NSCLC, with the most common types being exon 19 deletions, exon 21 p.L858R point mutations, and exon 20 insertions. These mutations have also been identified in BAs (2–4), and in this case, an EGFR exon 19 deletion was detected. ALK is a potent oncogenic driver, and ALK rearrangements, the second most common mutation after EGFR mutations in NSCLC, have also been observed in BAs (8, 9). The identification of driver mutations supports the clonal and neoplastic nature of BAs similar to adenocarcinoma. For patients with a confirmed diagnosis of BAs who are unable to undergo surgical intervention due to compromised health conditions or other reasons, whether gene targeted therapy drugs such as Osimertinib Mesylate Tablets, Crizotinib Capsules, gefitinib, etc. for lung cancer be considered for the treatment of BAs? Currently, there are no documented reports indicating the utilization of gene targete therapy drugs in the treatment of BAs, which represents a subject warranting in-depth investigation.

**Table 1:** Summary of previously reported gene mutations in BAs.

No	Author	Gene mutations (cases)				
1	Chang JC (2)	*BRAF V600E* (8)	*EGFR exon19 Del* (2)	*EGFR exon 20 insertion* (2)	*KRAS* (5)
2	Teng X (3)	*BRAF V600E* (5)			
3	Kamata T (7)	*BRAF V600E* (4)		*EGFR exon19 Del* (3)	
4	Yang C (4)	*EGFR exon 21 p.L858R* (1)			
5	Jin Y (8)	*ALK arrangement* (1)			
6	Taguchi R (9)	*ALK arrangement* (1)			
7	Liu L (5)	*BRAF V600E* (4)			
8	Emiko Udo (6)	*KRAS G12D* (1)		*BRAF V600E* (1)	

Given the bland cytology and lack of disease recurrence reported in many studies (15–17), BAs appear to represent benign adenomatous growths. However, the possibility of malignancy cannot be completely excluded due to certain features, such as destruction of the alveolar structure, central fibrosis, hyperplasia, lesions extending along the alveolar wall, micropapillary structures, loss of cell membrane integrity, and positive staining for CEA in immunohistochemical tests. Sato and Hata have classified BAs as low-grade malignant tumors (18, 19). Currently, most BAs are detected early by CT imaging and surgically removed, limiting observations of their biological behavior. Thus, no definitive conclusion has been reached regarding their nature.

During surgical treatment, intraoperative frozen sections are essential but are often misdiagnosed as adenocarcinoma for three main reasons. First, the diagnosis of BA relies on identifying a bilayered cellular structure within the tumor, particularly a complete basal cell layer. However, partial or complete loss of basal cells has been observed in some BAs, especially in distal types (2). Second, the histological features of well-differentiated small invasive adenocarcinoma and microinvasive adenocarcinoma closely resemble those of BAs in frozen sections. Third, BAs are prone to misdiagnosis with mucinous adenocarcinoma when there is a higher proportion of mucous components. Given these overlapping features, a definitive diagnosis can be challenging, even for experienced pathologists. In this case, the intraoperative frozen section suggested lung adenocarcinoma, but immunohistochemical examination ultimately confirmed the diagnosis of BA. Research of Ding (20) offers expertise in the identification of both diseases. First, BAs was often continuous with bronchi, while invasive adenocarcinoma usually did not continue with bronchi and may show destruction of bronchi. Second, The papillary architecture in BAs typically appears as round and smooth, whereas in adenocarcinoma it is characterized by a slender and rigid structure. Third, different with lung adenocarcinoma, the central region of the proximal-type BAs exhibits characteristics that are largely consistent with the surrounding areas, both in glandular and cellular features. Lastly, when a definitive diagnosis cannot be established, a descriptive diagnosis in the frozen section report should indicate the possibility of BA while acknowledging that small-volume invasive adenocarcinoma cannot be completely ruled out, for a more accurate diagnosis, immunohistochemical tests for P63, P40, CK5/6, and, along with careful evaluation of morphological features, should be considered.

## Conclusion

The pathological features of BAs, particularly distal types, resemble those of adenocarcinoma, often resulting in misdiagnosis during intraoperative frozen sections. A careful search for characteristics of BAs such as bilayer epithelial cells with basal cells and a lack of cellular atypia is warranted.

Additionally, BAs exhibit genetic mutations closely associated with lung adenocarcinoma. Whether BAs are entirely benign or possess malignant features remains under debate, and can gene targete therapy drugs such as Osimertinib Mesylate Tablets, Crizotinib Capsules, gefitinib, etc. for lung cancer be considered for the treatment of BAs. Further investigation is necessary.

## Data Availability

The original contributions presented in the study are included in the article/supplementary material. Further inquiries can be directed to the corresponding author.
